# A practical guide to bioelectrical impedance analysis using the example of chronic obstructive pulmonary disease

**DOI:** 10.1186/1475-2891-10-35

**Published:** 2011-04-21

**Authors:** Anja Walter-Kroker, Axel Kroker, Muriel Mattiucci-Guehlke, Thomas Glaab

**Affiliations:** 1 Nutritional Consulting Practice, Emil-Schüller-Straße, Koblenz, 56068, Germany; 2 Pneumology Practice, Emil-Schüller-Straße, Koblenz, 56068, Germany; 3 Medical Affairs Germany, Boehringer Ingelheim Pharma GmbH & Co. KG, Binger Straße, Ingelheim, 55218, Germany; 4 Department of Pulmonary Disease, III. Medical Clinic, Johannes Gutenberg-University, Langenbeckstraße, Mainz, 55131, Germany

**Keywords:** Bioelectrical impedance analysis, bioelectrical impedance vector analysis, malnutrition, chronic obstructive pulmonary disease

## Abstract

Bioelectrical impedance analysis (BIA) is a simple, inexpensive, quick and non-invasive technique for measuring body composition. The clinical benefit of BIA can be further enhanced by combining it with bioelectrical impedance vector analysis (BIVA). However, there is a substantial lack of information on the practical aspects of BIA/BIVA for those primarily interested in learning how to use and interpret this method in practice. The purpose of this article is to provide some guidance on the use of BIA/BIVA with special attention to practical considerations.

This report reflects the authors' practical experience with the use of single-frequency BIA in combination with BIVA, particularly in COPD patients. First, the method and principles of BIA/BIVA are briefly described. Then, a practice-oriented approach to the interpretation and analysis of characteristic examples of altered nutritional and fluid status as seen with BIA/BIVA in COPD patients (e.g. malnutrition in obese and underweight patients with COPD, water retention) is presented.

As our examples show BIA/BIVA is an attractive and easy-to-learn tool for quick nutritional assessment and is therefore of great clinical benefit in daily practice.

## Introduction

Loss of body weight and depletion of fat free muscle mass are common and serious problems in patients with chronic obstructive pulmonary disease (COPD) irrespective of the degree of airflow limitation [1-3]. Malnutrition in COPD has been associated with systemic inflammation, cachexia, anorexia, skeletal muscle dysfunction, dyspnoea, reduced health status, enhanced risk of exacerbations and increased mortality [4-8]. Consequently, current COPD guideline recommendations by GOLD (Global Initiative for Chronic Obstructive Lung Disease) consider nutritional monitoring an important part of routine evaluation of COPD patients [9].

Surrogate measures such as the well-known Body Mass Index (BMI) give no indication of body composition, muscle mass or nutritional state. Thus, malnutrition requiring intervention can exist in spite of a normal to high BMI. These patients are usually not detected by subjective global assessment of nutritional status. In the case of COPD, it has been recognized that it is low fat-free mass (FFM) further differentiated into body cell mass (BCM) and extra cellular mass (ECM) rather than low BMI that should be considered as a critical parameter of disease severity and prognosis [10,11].

Different methods are used for nutritional assessments beyond BMI, such as bioelectrical impedance analysis (BIA), skin-fold anthropometry and dual-energy X-ray absorptiometry (DEXA) [12-14]. BIA is a simple, inexpensive, quick and non-invasive technique for assessing body composition and its changes over time. BIA is largely used in clinical trial settings and there is a whole series of literature on the theory and methodology of several different BIA techniques [15-18]. Surprisingly enough, however, there is considerable lack of information on the practical aspects of BIA for those primarily interested in learning how to apply and interpret this method in practice. Thus, BIA still is an underused and underestimated tool for nutritional assessment in primary care. This can be further explained by the fact that the costs of BIA are currently not always refundable [12] and that there are no guidelines outlining the methods for assessing malnutrition in patients with COPD [9,19].

BIA analysis is simply and effectively complemented by bioelectrical impedance vector analysis (BIVA), which is independent of hydration status and can be used as a quality control measure for correct interpretation of BIA results [20,21]. BIVA is a pattern analysis of impedance measurements (resistance and reactance) plotted as a vector in a coordinate system [21]. Reference values adjusted for age, BMI and gender are plotted as so-called tolerance ellipses in the coordinate system. On this basis, a statement can be made with regard to water balance (normo-, hypo-, hyperhydration) and body cell mass (nutritional status) [14].

The purpose of this article is to provide some guidance on the use of BIA/BIVA with particular attention to practical considerations. It reflects a decade of our own practical experience of using single-frequency BIA combined with BIVA with a focus on COPD patients. Here, we briefly describe the basic principles, feasibility and limitations of BIA/BIVA methodology and provide practical tips and recommendations for appropriate conduct and analysis of the measurements. We have aimed to provide a simple, structured and practice-oriented approach to the interpretation and analysis of BIA/BIVA results using characteristic examples seen in patients with COPD.

### Basic principles

#### Bioelectrical impedance analysis (BIA)

BIA is a method for estimating body composition. The principle of BIA is to determine the electric impedance of an electric current passing through the body [15]. The electrical impedance (Z) consists of two components, resistance (R) and reactance (Xc). Reactance is a measure of BCM and resistance a measure of total body water [15,22]. From the determined impedance a number of BIA parameters can be estimated [20]:

#### Body cell mass (BCM)

• consists of all cells that have an effect on metabolism (e.g. muscle, internal organs, nervous system)

• key factor when assessing the nutritional status of a patient

• ↑: e.g. good training status, intracellular water retention

• ↓: e.g. malnutrition, cachexia, dehydration

#### BCM %

• percentage of BCM in FFM

• for differential diagnosis of BIA changes: changes in the same way as BCM

• measure of individual nutritional status and physical fitness level

• ↑: e.g. good training status

• ↓: e.g. malnutrition

#### Extra cellular mass (ECM)

• mainly extracellular water

• increase or decrease mostly due to increased extracellular water retention or a loss of extracellular water

• ↑: e.g. extracellular water retention (e.g. oedema)

• ↓: e.g. extracellular loss of water (e.g. diuretics)

#### Fat-free mass (FFM)

• everything that is not body fat, consists of BCM and ECM

• ↓: elderly people, chronic diseases

#### Fat mass (FM)

• is indirectly determined from body weight minus FFM

#### Phase angle

• one of the best indicators of cell membrane function

• can be regarded as a marker of training and nutritional status

• ↑: e.g. athletic constitutional type, good nutritional status (of BCM cells)

• ↓: e.g. poor training status, poor nutritional status (of BCM cells)

#### Total body water (TBW)

• ↑: e.g. high portion of muscle, water retention (e.g. oedema)

• ↓: e.g. small portion of muscle, dehydration/exsiccosis

The strengths and limitations of different BIA methods (e.g. single frequency, multi-frequency, segmental BIA) have been extensively reviewed [15,16,18,23-25].

Our experience is based on single frequency BIA (50 kHz); the software package we use (NUTRIPLUS from Data Input GmbH) includes BIVA and adapted reference values.

Detailed instructions for performing BIA measurements can be found elsewhere [17,18]. To give a brief description here, single frequency BIA usually involves the placing of two distal current or signal-introducing electrodes on the dorsal surfaces of the hand and foot close to the metacarpal-phalangeal and metatarsal-phalangeal joints, respectively. The two voltage sensing electrodes are applied at the pisiform prominence of the wrist and between the medial and lateral maleoli of the ankle. The impedance analyzer delivers a constant alternating current at a fixed 50-kHz frequency via the distal electrodes and detects the drop in voltage via the proximal electrodes. The measured resistance and reactance are displayed by the analyzer [18].

Factors impacting BIA results [16,18,20,23,25]:

1. weight and height (should be measured directly by the investigator)

2. position of the body and limbs (supine position, arms abducted at least 30°, legs abducted at approximately 45°)

3. consumption of food and beverages (no beverages for at least 12 hours previously, fasted state for at least 2 hours)

4. moderate to intense level of physical activity/exercise before BIA measurements (last exercise at least 12 hours previously)

5. medical conditions and medication that have an impact on the fluid and electrolyte balance; infection and cutaneous disease that may alter the electrical transmission between electrode and skin

6. environmental conditions (e.g. ambient temperature)

7. individual characteristics (e.g. skin temperature, sex, age, race)

8. ethnic variation

9. non-adherence of electrodes, use of wrong electrodes, loosening of cable clip, interchanging of electrodes

BIA parameters are largely dependent on the patient's hydration status. BIA enables the above mentioned parameters to be determined in subjects without significant fluid and electrolyte abnormalities [15].

### BIVA (bioelectrical impedance vector analysis)

**Figure 1:**
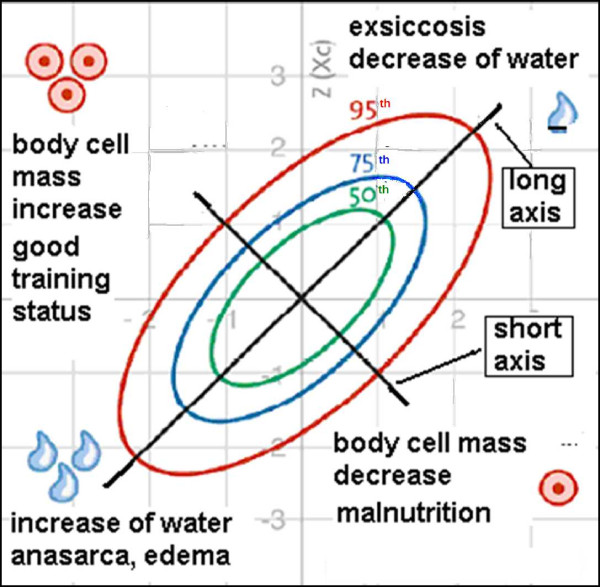
**Interpretation of the BIVA nomogram**. Age, BMI and gender adjusted reference values are plotted as so-called tolerance ellipses in the coordinate system. Three tolerance ellipses are distinguished, corresponding to the 50^th^, 75^th ^and 95^th ^vector percentile of the healthy reference population. Values outside of the 95^th ^percentile are considered abnormal. Values located above the long axis (/) indicate an increase in body cell mass (BCM), values below the long axis indicate a decrease in BCM. Values located above the short axis (\) indicate a loss of water and values below the short axis indicate increased water retention. Values located outside the 95^th ^percentile in the following four quadrants point to the following conditions: a) right upper quadrant e.g. exsiccosis b) left lower quadrant e.g. oedema c) right lower quadrant e.g. malnutrition d) left upper quadrant e.g. good training status (modified with permission from Data-Input GmbH).

BIVA as an integrated part of BIA measurement is a simple, quick and clinically valuable method for assessing fluid status (TBW) and body cell mass (BCM). This method plots the direct impedance measurements resistance R and reactance Xc as a bi-variate vector in a nomogram (Figure 1) [21]. Reference values adjusted for age, BMI and gender are plotted as so-called tolerance ellipses in the same coordinate system. Three tolerance ellipses are distinguished, corresponding to the 50^th^, 75^th ^and 95^th ^vector percentile of the healthy reference population [22,26]. Values outside of the 95^th ^percentile are considered abnormal. Based on the position of the measurement point in the BIVA nomogram, the sex-, age-, BMI- and race-adjusted nutrition/training and hydration status can be read off at a glance [21]. As shown in Figure 1, values located outside the 95^th ^percentile in the following four quadrants point to the following conditions [20]: a) right upper quadrant e.g. exsiccosis b) left lower quadrant e.g. oedema c) right lower quadrant e.g. malnutrition d) left upper quadrant e.g. good training status.

### Applications of BIA/BIVA and interpretation of body composition

We present below some examples of characteristic BIA findings in COPD patients with their interpretation:

1. Normal finding

2. Malnutrition in a COPD patient with overweight

3. Cachexia

4. Oedema due to right heart failure

5. Anorexia

From personal experience, follow-up measurements (examples 2-5) should be performed every 4 weeks for overweight patients and every 8-12 weeks for all other cases [27]. However, this is a decision that must be taken on an individual basis.

#### 1. Normal finding

Patient: female, 61.1 kg, BMI 22.7, 64 years old, mild COPD (GOLD stage I) [9].

**Figure 2:**
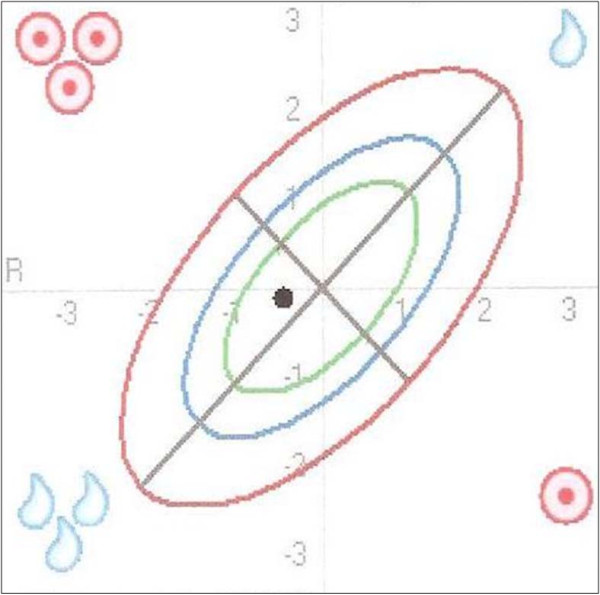
**Normal finding as illustrated in the BIVA nomogram**. The position of the measurement point in the BIVA nomogram within the 50^th ^tolerance ellipse (range of normal values) indicates a normal finding.

Interpretation: With a BMI of 22.7 this patient is of normal weight. The measurement point in the BIVA nomogram (Figure 2) lies within the 50^th ^tolerance ellipse and thus indicates normal findings. The TBW, ECM, body fat, BCM, BCM % and phase angle values listed in table 1 are within the normal range.

**Table 1:** Normal finding.

parameter	values	
TBW	33.2 l	[29.1 - 36.1 l]
FFM	45.4 kg	[39.8 - 49.4 kg]
ECM	22.7 kg	[19.5 - 25.7 kg]
BCM	22.7 kg	[18.8 - 25.3 kg]
BCM %	50.1 %	[44.7 - 53.9 %]
phase angle	5.6 °	[4.7 - 6.4 °]
body fat in kg (corrected)	15.6	[13.3 - 23.7 kg]

Values for different BIA parameters. Age- and BMI-adapted reference values are shown in brackets. All values are within the normal range.

Conclusion: All values in the table are within the normal range and the measurement point in the BIVA nomogram lies within the 50^th ^tolerance ellipse.

#### 2. Malnutrition in an obese COPD patient

Patient: female, 90 kg, BMI 31.5, 73 years old, severe COPD (GOLD stage III)

**Figure 3:**
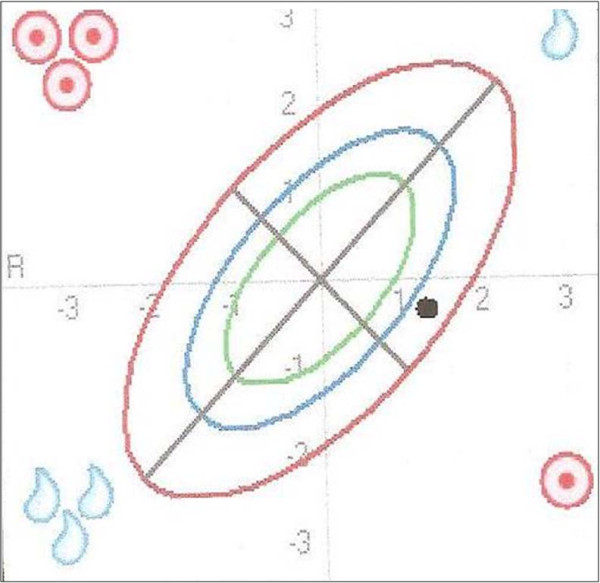
**Malnutrition in an obese COPD patient as illustrated in the BIVA nomogram**. The position of the measurement point in the BIVA nomogram is below the line of normal BCM values (long axis) and above the line of normal TBW values (short axis) between the 75^th ^and 95^th ^tolerance ellipse. The position in the lower right quadrant indicates malnutrition.

Interpretation: With a BMI of 31.5 this patient is obese. The measurement point in the BIVA nomogram (Figure 3) in this patient is well below the line of normal BCM values (long axis) and above the line of normal TBW values (short axis) between the 75^th ^and the 95^th ^tolerance ellipse. The position of the measurement point in the lower right quadrant points to malnutrition.

The BIA parameter values listed in table 2 can be interpreted as follows: The fat mass lies above the normal range in line with the increased BMI. BCM lies within the normal range. At first sight this does not fit in with the finding of the BIVA nomogram, which indicates malnutrition. The fact that the calculated BCM is within the range of normal values here may be explained as follows: It needs to be considered that BCM is dependent on the patient's fluid status (TBW). This means that a BCM within the normal range does not necessarily mean a normal nutritional status but may also be due to increased TBW. The following two BIA parameters are helpful in drawing a distinction: BCM % and phase angle, which are usually altered along the same lines as BCM. In this case TBW is increased and the BCM % and phase angle are below the range of normal values. This indicates that BCM is actually reduced. BCM therefore only appears to lie within the range of normal values because of the increased TBW. In contrast to this somewhat complex interpretation of the calculated BIA values, the suspected diagnosis of malnutrition can be established at a glance by BIVA. In addition, it is confirmed that the calculated BCM is too high because of the increased TBW (position of the measurement point in the BIVA nomogram above the line of normal TBW values).

**Table 2:** Malnutrition in an obese COPD patient

parameter	values	
TBW	**36.7 l**	[29.1 - 36.1 l]
FFM	**50.1 kg**	[39.8 - 49.4 kg]
ECM	**29.1 kg**	[19.5 - 25.7 kg]
BCM	21.0 kg	[18.8 - 25.3 kg]
BCM %	**42.0 %**	[44.7 - 53.9 %]
phase angle	**4.3 °**	[4.7 - 6.4 °]
body fat in kg (corrected)	**39.9 kg**	[13.3 - 23.7 kg]

Values for different BIA parameters. Age- and BMI-adapted reference values are shown in brackets. Bold values are outside the normal range.

Conclusion: Despite the presence of obesity the patient is exhibiting malnutrition. The position of the measurement point in the BIVA nomogram in the right lower quadrant between the 75^th ^and the 95^th ^tolerance ellipse provides an indication for the suspected diagnosis of malnutrition.

#### 3. Cachexia

Patient: male, 45 kg, BMI 16.7, 62 years old, severe COPD (GOLD stage III)

**Figure 4:**
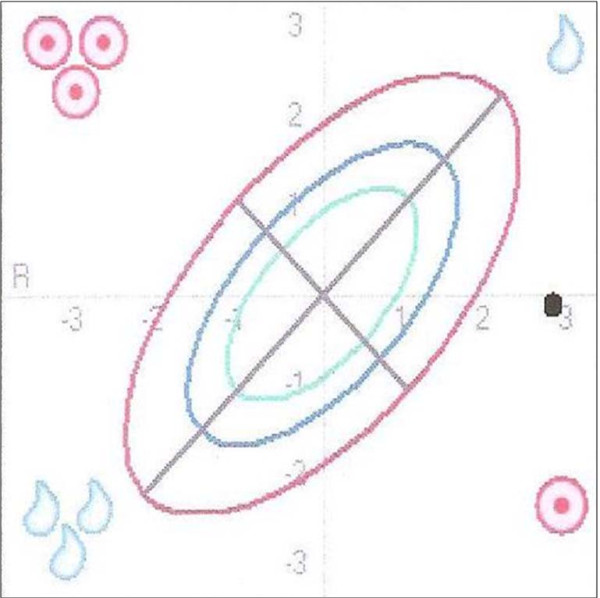
**Cachexia as illustrated in the BIVA nomogram**. The position of the measurement point in the BIVA nomogram is far below the line of normal BCM values (long axis) and well above the line of normal TBW values (short axis) far outside the 95^th ^tolerance ellipse. The position in the lower right quadrant points to cachexia.

Interpretation: With a BMI of 16.7 the patient is underweight. The measurement point in the BIVA nomogram (Figure 4) in this patient is far below the line of normal BCM values (long axis) and well above the line of normal TBW values (short axis), far outside the 95^th ^tolerance ellipse. The position of the measurement point in the lower right quadrant points to malnutrition in the form of cachexia.

The BIA parameter values listed in table 3 can be interpreted as follows: The fat mass lies below the normal range in line with the reduced BMI. The calculated values for BCM und TBW are reduced. It needs to be considered as regards the reduced BCM value that BCM is dependent on the patient's fluid status (TBW). This means that a reduced BCM does not necessarily point to malnutrition but may also be due to a low TBW. The BIA parameters BCM % and phase angle are again helpful for drawing a distinction, as they are usually altered along the same lines as BCM. The values for BCM % and phase angle are reduced, which indicates an actually reduced BCM or malnutrition. In this example also BIVA provides a more efficient assessment of the nutritional status than the calculated BIA parameters.

**Table 3:** Cachexia

parameter	values	
TBW	**29.1 l**	[37.6 - 48.4 l]
FFM	**39.8 kg**	[51.4 - 66.1 kg]
ECM	**22.5 kg**	[24.6 - 33.7 kg]
BCM	**17.3 kg**	[25.1 - 34.0 kg]
BCM %	**43.5 %**	[44.7 - 55.0 %]
phase angle	**4.5 °**	[4.7 - 6.6 °]
body fat in kg (corrected)	**5.2 kg**	[7.9 - 20.1 kg]

Values for different BIA parameters. Age- and BMI-adapted reference values are shown in brackets. All values are below the normal range.

Conclusion: All the values listed in the table are below the normal range and the measurement point in the BIVA nomogram is outside the 95^th ^tolerance ellipse in the lower right quadrant. This indicates severe malnutrition in the form of cachexia. The assessment of the BIVA nomogram is sufficient for the suspected diagnosis of cachexia.

#### 4. Oedema due to right heart failure

Patient: female, 105.6 kg, BMI 38.8, 78 years old, COPD GOLD stage II

**Figure 5:**
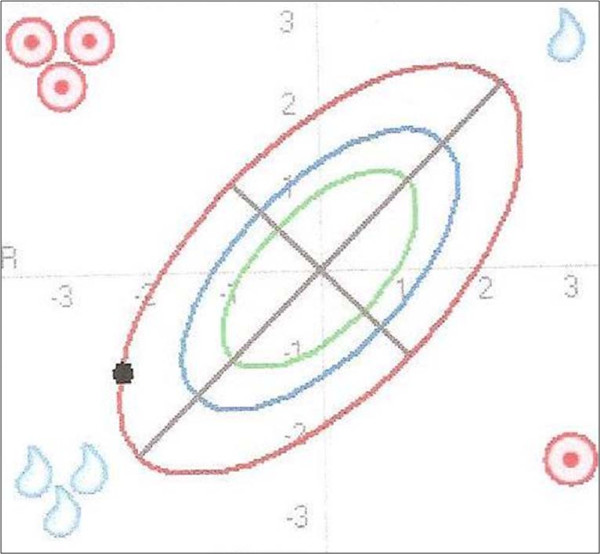
**Oedema due to right heart failure as illustrated in the BIVA nomogram**. The position of the measurement point in the BIVA nomogram is above the line of normal BCM values (long axis) and well below the line of normal TBW values (short axis) on the 95^th ^tolerance ellipse. The position in the lower left quadrant indicates the presence of increased water retention.

Interpretation: With a BMI of 38.8 the patient is overweight. The measurement point in the BIVA nomogram (Figure 5) in this patient is above the line of normal BCM values (long axis) and well below the line of normal TBW values (short axis) on the 95^th ^tolerance ellipse. The position of the measurement point in the lower left quadrant points to water retention in the form of oedema.

The BIA parameter values listed in table 4 can be interpreted as follows: Body fat mass lies above the normal range in line with the increased BMI. The determined TBW is increased and the calculated BCM lies in the upper range of normal. These findings are consistent with the position of the measurement point above the line of normal BCM values and below the line of normal TBW values in the lower left quadrant. With the derived normal BIA value for BCM it needs once again to be taken into account here that BCM is dependent on the patient's fluid status (TBW). This means that a BCM within the normal range does not necessarily indicate an actually normal BCM or normal nutritional status but may also appear normal due to an increased TBW. The BIA parameters BCM % and phase angle, which are usually altered along the same lines as BCM, in this case lie in the lower range of normal, so that it may be assumed that the BCM is actually in the normal range. In addition to the increased TBW, ECM is also markedly increased, indicating oedema. The suspicion of oedema is established at a glance with BIVA. BIVA confirms simply and rapidly the calculated BIA values BCM and TBW. The suspicion of oedema was confirmed on physical examination of the legs.

**Table 4:** Oedema due to right heart failure

Parameter	values	
TBW	**49.3 l**	[36.1 - 45.1 l]
FFM	**67.3 kg**	[49.3 - 61.6 kg]
ECM	**34.5 kg**	[23.0 - 30.6 kg]
BCM	32.8 kg	[24.6 - 32.8 kg]
BCM %	48.7 %	[47.2 - 56.0 %]
phase angle	5.4 °	[5.1 - 6.9 °]
body fat in kg (corrected)	**40.2 kg**	[22.7 - 35.0 kg]

Values for different BIA parameters. Age- and BMI-adapted reference values are shown in brackets. Bold values are outside the normal range.

Conclusion: The values listed in the table for TBW and ECM are outside the normal range and the measurement point in the BIVA nomogram is on the 95^th ^tolerance ellipse in the lower left quadrant, indicating oedema. The determined BCM is in the upper range of normal and the measurement point in the BIVA nomogram is above the line of normal BCM values. The position of the measurement point in the nomogram provides an indication for the suspected diagnosis of oedema.

#### 5. Anorexia

For the general differential diagnosis of underweight we present a female patient with anorexia: female, 34.1 kg, BMI 18.4, 41 years old, anorexia (unintentional loss of appetite).

**Figure 6:**
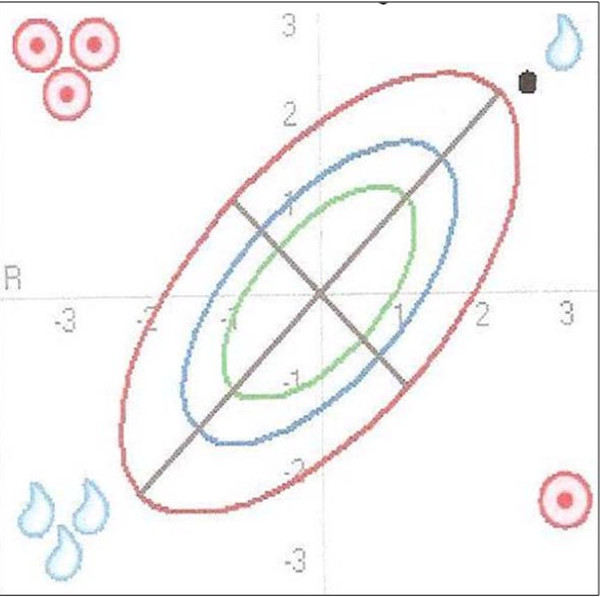
**Anorexia as illustrated in the BIVA nomogram**. The position of the measurement point in the BIVA nomogram is almost on the line of normal BCM values (long axis) and far above the line of normal TBW values (short axis) outside the 95^th ^tolerance ellipse. The position in the upper right quadrant points to the presence of anorexia.

Interpretation: With a BMI of 18.4 the patient is underweight. The measurement point in the BIVA nomogram (Figure 6) lies almost on the line of normal BCM values (long axis) and far above the line of normal TBW values (short axis) outside the 95^th ^tolerance ellipse. The position of the measurement point in the upper right quadrant points to the presence of anorexia.

The BIA parameter values listed in table 5 can be interpreted as follows: Body fat mass is reduced in line with the low BMI. TBW is markedly reduced and BCM also is decreased. With the reduced BCM it needs to be kept in mind here that BCM is dependent on the patient's fluid status (TBW). This means that a lower BCM may also appear reduced due to a lower TBW. The BIA parameters BCM % and phase angle which are helpful in drawing a distinction and which are usually altered in the same way as BCM, in this case lie in the upper third of the normal range. This indicates that BCM is normal and that the calculated value was too low only because of the low TBW. BIVA confirms the suspicion raised by the BIA values that the calculated BCM was too low because of the reduced TBW. Again, the suspected diagnosis of anorexia can be established more efficiently and more reliably by BIVA.

**Table 5:** Anorexia

parameter	values	
TBW	**20.1 l**	[29.9 - 36.9 l]
FFM	**27.4 kg**	[40.8 - 50.4 kg]
ECM	**12.9 kg**	[19.1 - 25.0 kg]
BCM	**14.5 kg**	[20.4 - 26.6 kg]
BCM %	53 %	[47.7 - 55.4 %]
phase angle	6.2 °	[5.2 - 6.8 °]
body fat in kg (corrected)	**6.7 kg**	[13.9 - 24.2 kg]

Values of the different BIA parameters. Age- and BMI-adapted reference values are shown in brackets. Bold values are outside the normal range.

Conclusion: The patient exhibits a markedly reduced BMI, decreased body water and a normal BCM in the form of anorexia. The position of the measurement point in the nomogram in the upper right quadrant outside the 95^th ^tolerance ellipse provides an indication for the suspected diagnosis of anorexia.

## Summary

Bioelectrical impedance analysis (BIA), particularly in combination with bioelectrical impedance vector analysis (BIVA), provides a viable opportunity for evaluating body composition in humans. However, lack of guidance for those interested in learning how to use and interpret BIA/BIVA in clinical practice has probably prevented its broader application. This practical guidance which is mainly based on own practical experience has attempted to provide a direction on the use of BIA/BIVA methodology with particular attention given to practical considerations. The basic principles of performing BIA/BIVA measurements have been given as well as characteristic examples in COPD patients.

As the examples suggest the interpretation of BIA results is often complex and a suspected diagnosis can be established more efficiently and more reliably by integrating BIVA into the patient assessment process. Although this review was not intended to be comprehensive and is not evidence-based, it has hopefully provided a brief description of some of the practicalities involved in performing BIA/BIVA measurements and, perhaps more importantly, has raised interest in actively using BIA/BIVA in daily clinical practice.

## Abbreviations

ATS: American Thoracic Society; BCM: body cell mass; BIA: bioelectrical impedance analysis; BIVA: bioelectrical impedance vector analysis; BMI: body mass index; COPD: chronic obstructive pulmonary disease; ECM: extra cellular mass; ERS: European Respiratory Society; FFM: fat free mass; FM: fat mass; GOLD: Global Initiative for Chronic Obstructive Lung Disease; TBW: total body water.

## Competing interests

The authors declare that they have no competing interests. TG and MMG were employees of Boehringer Ingelheim at the time of manuscript submission.

## Authors' contributions

AWK and TG conceived of the review, drafted and coordinated the manuscript. MMG and AK critically discussed and helped to draft the manuscript. All authors read and approved the final manuscript. The contents of this original manuscript have not been previously presented or submitted elsewhere.
